# Expansion of *Neisseria meningitidis* Serogroup C Clonal Complex 10217 during Meningitis Outbreak, Burkina Faso, 2019

**DOI:** 10.3201/eid3003.221760

**Published:** 2024-03

**Authors:** Joann F. Kekeisen-Chen, Felix T. Tarbangdo, Shalabh Sharma, Daya Marasini, Henju Marjuki, Janelle L. Kibler, Heather E. Reese, Seydou Ouattara, Flavien H. Ake, Issaka Yameogo, Issa Ouedraogo, Emmanuel Seini, Robert L. Zoma, Issa Tonde, Mahamoudou Sanou, Ryan T. Novak, Lucy A. McNamara

**Affiliations:** Centers for Disease Control and Prevention, Atlanta, Georgia, USA (J.F. Kekeisen-Chen, S. Sharma, D. Marasini, H. Marjuki, J.L. Kibler, H.E. Reese, R.T. Novak, L.A. McNamara);; Davycas International, Ouagadougou, Burkina Faso (F.T. Tarbangdo, F.H. Ake, R.L. Zoma);; Ministère de la Santé, Ouagadougou (S. Ouattara, I. Yameogo, I. Ouedraogo, E. Seini);; Centre Hospitalier Universitaire Pédiatrique Charles de Gaulle, Ouagadougou (I. Tonde, M. Sanou);; Université Joseph Ki-Zerbo, Ouagadougou (I. Tonde, M. Sanou)

**Keywords:** Neisseria meningitidis, Africa meningitis belt, serogroup C, clonal complex 10217, sequence type, vaccination, bacteria, meningitis/encephalitis, Burkina Faso

## Abstract

During January 28–May 5, 2019, a meningitis outbreak caused by *Neisseria meningitidis* serogroup C (NmC) occurred in Burkina Faso. Demographic and laboratory data for meningitis cases were collected through national case-based surveillance. Cerebrospinal fluid was collected and tested by culture and real-time PCR. Among 301 suspected cases reported in 6 districts, *N. meningitidis* was the primary pathogen detected; 103 cases were serogroup C and 13 were serogroup X. Whole-genome sequencing revealed that 18 cerebrospinal fluid specimens tested positive for NmC sequence type (ST) 10217 within clonal complex 10217, an ST responsible for large epidemics in Niger and Nigeria. Expansion of NmC ST10217 into Burkina Faso, continued NmC outbreaks in the meningitis belt of Africa since 2019, and ongoing circulation of *N*. *meningitidis* serogroup X in the region underscore the urgent need to use multivalent conjugate vaccines in regional mass vaccination campaigns to reduce further spread of those serogroups.

Burkina Faso is a landlocked country within the meningitis belt of sub-Saharan Africa that experiences hyperendemic bacterial meningitis and an elevated risk for recurrent meningitis outbreaks (*1*). Commonly characterized by headache, fever, stiff neck, and altered consciousness, bacterial meningitis can lead to permanent disability or death if not quickly detected and treated.

Historically, meningitis epidemics within the meningitis belt of Africa have been caused primarily by *Neisseria meningitidis* serogroup A (NmA) (*2*–*4*). In 2010, Burkina Faso was the first of many meningitis-belt countries to introduce the novel monovalent meningococcal serogroup A conjugate vaccine, MenAfriVac, nationwide. After MenAfriVac introduction, meningitis cases and outbreaks caused by NmA were no longer reported (*2*,*4*). However, seasonal meningitis outbreaks and epidemics still occur in the region because of non-NmA serogroups, including *N. meningitidis* serogroup C (NmC), X (NmX), and W (*5*). In particular, NmC has caused several large outbreaks both within and outside the meningitis belt in the past several years (*6*).

Meningitis caused by NmC had been generally uncommon in the meningitis belt of Africa. Occasional NmC outbreaks and epidemics have been reported in the belt during the past 50 years, including an NmC epidemic in 1975 in northern Nigeria and small, localized outbreaks in Burkina Faso in 1979, Mali during 1988–1992, and Nigeria during 2013–2014 (*7*–*11*). In 2015, however, NmC emerged as a serious public health threat after a focal NmC infection outbreak in northern Nigeria spread to neighboring Niger, where a large NmC epidemic that had 9,367 suspected cases and 549 deaths was reported (*12*). In 2017, the largest recorded meningitis epidemic caused by NmC occurred in northern Nigeria; 14,518 suspected cases and 1,166 deaths were reported (*13*). Through molecular typing, the recent epidemics in Niger and Nigeria were shown to be caused by a new NmC strain, sequence type (ST) 10217 belonging to clonal complex (CC) 10217 (*12*,*14*). Comparative genomic analysis suggested that NmC ST10217 emerged from a meningococcal strain previously identified in nasopharyngeal specimens of asymptomatic human carriers after acquiring virulence genes (*15*).

In January 2019, a cluster of unexplained deaths was reported from the Boutou commune of Diapaga District in Burkina Faso’s Est administrative region. The Est region shares its northern border with Niger and southern border with Benin and Togo. The chief medical officer, regional director of health, and director of population health protection were alerted, which led to epidemiologic investigations in Diapaga that confirmed a meningitis outbreak caused by NmC. Because of the rise of NmC cases in neighboring countries, recent large NmC outbreaks in the region, concern for the spread of NmC ST10217, and limited availability of NmC vaccines, outbreak investigation was critical to elucidate the evolving epidemiology of meningitis within the region (*16*). We describe the 2019 meningitis outbreak in Burkino Faso, the outbreak response, and microbiologic features of the NmC strain driving the outbreak.

## Methods

### Meningitis Surveillance

Population-based meningitis surveillance exists in 2 complementary systems in Burkina Faso (*17*). First, district-level aggregate reports of clinically defined (suspected) meningitis cases and meningitis-related deaths are transmitted weekly by the Telegramme Lettre Official Hebdomadaire (TLOH). The TLOH system has been functional in Burkina Faso since 1997 but does not hold any laboratory or demographic information aside from that obtained from the administrative district reporting cases (*17*). Second, the TLOH system is complemented by nationwide case-based surveillance (CBS), conducted by using the cloud-based System for Tracking Epidemiologic Data and Laboratory Specimens (STELAB). STELAB collects detailed case-level demographic, clinical, and laboratory data and assigns barcodes to each case report form and collected specimen, enabling real-time tracking of a specimen’s journey from the district laboratory to the regional laboratory, then finally to a national reference laboratory (NRL) (*1*). National meningitis CBS data collected during 2018–2020 were validated in June 2021 and used for this analysis.

Alert and epidemic thresholds were defined according to published World Health Organization (WHO) guidelines. The alert threshold was defined as >3 suspected cases per week/100,000 inhabitants; the epidemic threshold was defined as >10 suspected cases per week/100,000 inhabitants (*18*). A suspected case was defined according to WHO guidelines as a sudden onset of fever (>38.5°C) accompanied by neck stiffness, altered consciousness, or other meningeal signs, including flaccid neck, bulging fontanelle, or convulsions in children <2 years of age (*18*). A confirmed bacterial meningitis case was defined as any suspected or probable case that was laboratory confirmed by culturing or by identifying a bacterial pathogen (*N. meningitidis*, *Streptococcus pneumoniae*, *Haemophilus influenzae type b*) in the cerebrospinal fluid (CSF) or blood by PCR as previously described (*18*).

### Laboratory

CSF specimens were collected from patients with suspected meningitis as part of routine surveillance. Confirmatory testing and serogrouping were performed by direct real-time PCR at the NRL. We performed further serogroup confirmation and molecular characterization for 18 CSF specimens at the Bacterial Meningitis Laboratory, National Center for Immunization and Respiratory Diseases, Centers for Disease Control and Prevention (CDC) (Atlanta, GA, USA). We enriched the specimens by using selective whole-genome amplification procedures and assessed the amplification by real-time PCR of the superoxide dismutase gene, *sodC*, as previously described (*19*). We performed whole-genome sequencing of all 18 CSF specimens that yielded a PCR cycle threshold of <16 for *sodC* after enrichment; the resulting genome assembly containing >1,400 core-genome multilocus sequence typing loci (*20*). We analyzed sequencing data by using the analysis pipeline developed in-house (*19*). We determined clonal complex and sequence types and characterized the gene locus encoding the polysaccharide capsule and peptide typing loci as previously described (*19*). For *N. meningitidis* CC10217 phylogenetic analysis, we compared 278 high-quality genome assemblies from isolates collected from Africa during 2012–2019 and the 18 Burkina Faso outbreak samples from 2019. All whole-genome sequencing data from this outbreak are publicly available in the PubMLST database (https://pubmlst.org/neisseria) (Appendix Table).

### Epidemiologic Analyses

We calculated cumulative incidence as the number of reported suspected meningitis cases in CBS data per 100,000 inhabitants by using 2019 health district population data. District populations in 2019 were provided as part of the TLOH line list by the Burkina Faso Ministry of Health. We extracted details of events prompting outbreak investigations from investigative reports provided by health districts under the supervision of the Ministry of Health. We determined the timing of key events related to the outbreak according to WHO weekly meningitis surveillance bulletin reports derived from TLOH data during the period of interest. We then analyzed CBS data in parallel to confirm the chronology of events and provide case-level laboratory and demographic information for each suspected case. We collected dates, administrative coverage, and vaccine type for reactive vaccination campaigns in each affected district from vaccination campaign reports provided by the Direction de la Protection de la Santé de la Population under Burkina Faso’s Ministry of Health.

**Figure 1:**
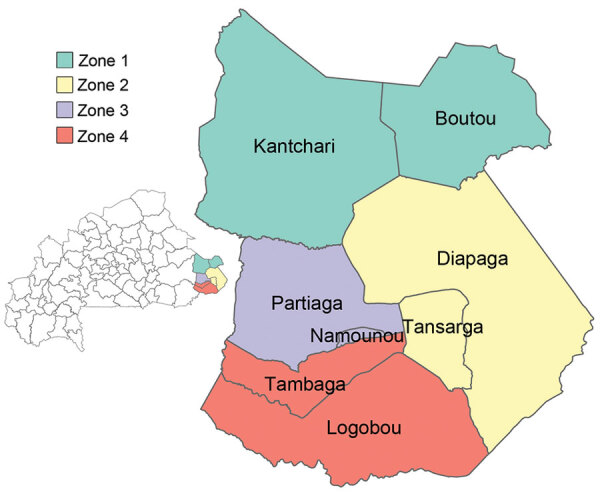
Meningitis surveillance zones in study of expansion of *Neisseria meningitidis* serogroup C clonal complex 10217 during meningitis outbreak, Burkina Faso, January 28–May 5, 2019. Main map indicates Diapaga health district divided into 8 communes within Tapoa Province of Burkina Faso. Inset map indicates the location of Tapoa Province in Burkina Faso. Colors indicate subdivision of the health district into 4 epidemiologic surveillance zones that have ≈100,000 inhabitants per zone.

We used shapefiles of the national, regional, and health district boundaries obtained from the Direction de la Protection de la Santé de la Population to show the geographic distribution of cases during the outbreak. We defined the spatial location of cases as the patient’s reported district of residence. The Diapaga health district is divided into 8 communes and has 37 health facilities that each serve a population covering ≈11 km^2^ of land. For surveillance purposes, the district has been subdivided into 4 epidemiologic surveillance zones that have ≈100,000 inhabitants per zone (Figure 1). For our analysis, we mapped Diapaga’s zones according to each commune’s corresponding health facility zoning in 2019. We maintained datasets and analytic results in Microsoft Excel version 2108 (https://www.microsoft.com) and performed data analyses and mapping by using R version 4.1.3 (The R Project for Statistical Computing, https://www.r-project.org). This work was reviewed by CDC and conducted consistent with applicable federal law and CDC policy (e.g., 45 Code of Federal Regulation part 46, 21 Code of Federal Regulation part 56; 42 United States Code [U.S.C.] §241(d); 5 U.S.C. §552a; 44 U.S.C. §3501 et seq.).

## Results

### Outbreak and Response Timelines

During January 28–January 31, 2019, a total of 19 suspected cases (zone 1, 17 cases; zone 2, 2 cases) were reported from Diapaga; CSF samples were collected from each patient (Figure 1). On January 31, PCR testing was performed on 12 of 19 specimens; 7 were confirmed positive for NmC. The remaining 7 samples were tested on February 11, confirming 3 additional NmC cases. All 10 confirmed cases came from zone 1. A reactive vaccination campaign was conducted during February 9–13 in 11 health facilities located within zone 1 of Diapaga by using the national stockpile of plain polysaccharide MenACWY vaccine. The campaign targeted persons who were 2–29 years of age and achieved an estimated 108% administrative coverage of the targeted population. No additional confirmed meningitis cases caused by any pathogen were reported from zone 1 during the 2019 epidemic season after February 11.

**Figure 2:**
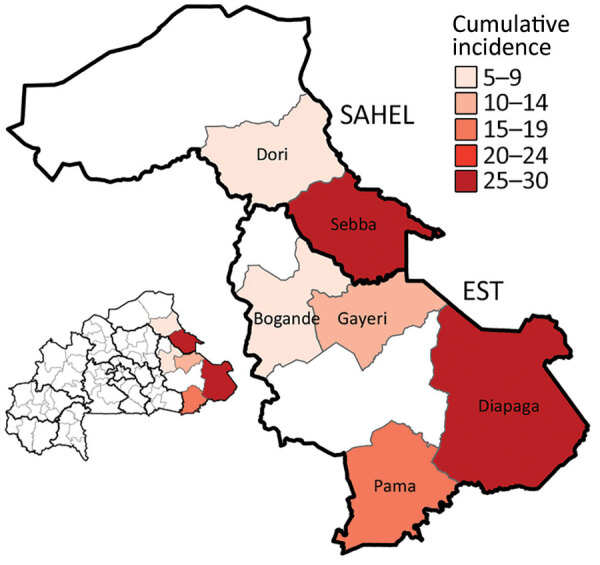
Meningitis cumulative incidence (cases/100,000 population) across 6 affected districts in the Sahel and Est regions of Burkina Faso in study of expansion of *Neisseria meningitidis* serogroup C clonal complex 10217 during meningitis outbreak, January 28–May 5, 2019. Colors indicate incidence of meningitis cases per 100,000 population. Inset map shows location of surveilled regions in Burkina Faso.

**Figure 3:**
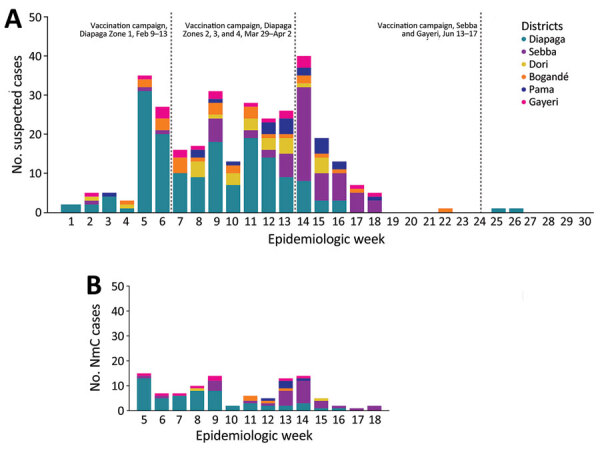
Number of meningitis cases in 6 affected districts in study of expansion of NmC clonal complex 10217 during meningitis outbreak, Burkina Faso, January 28–May 5, 2019. Colors indicate the specific district in Burkina Faso. A) Numbers of reported meningitis cases (suspected, probable, and confirmed), January 1–June 31, 2019. B) Numbers of confirmed NmC cases, January 28–May 5, 2019. NmC, *Neisseria meningitidis* serogroup C.

From the end of February through April, the outbreak spread to Diapaga zones 2, 3, and 4 and to neighboring districts (Pama in the Est region and Sebba and Gayeri in the Sahel region) (Figures 1, 2). A vaccine request was submitted to WHO’s International Coordinating Group (ICG) on Vaccine Provision on March 8; vaccines were delivered to affected areas on March 27 and, during March 29–April 2, a second vaccination campaign with conjugate MenACWY vaccines obtained through ICG was conducted in health facilities within Diapaga zones 2, 3, and 4 (Figure 3, panel A). The campaign targeted persons 1–29 years of age and achieved an estimated 111% administrative coverage. An additional ICG vaccine request was submitted on April 14 to cover Sebba and Gayeri. Plain polysaccharide MenACW vaccines were obtained through ICG, and a third campaign targeting persons 2–29 years of age was conducted during June 13–17, achieving an estimated 80% administrative coverage in Sebba and 87% in Gayeri (Figure 3, panel A). The second and third reactive vaccination campaigns were conducted in locations that either crossed the epidemic threshold (Diapaga zone 2, Sebba) or were considered to be at risk for outbreak expansion (Diapaga zones 3 and 4, Gayeri).

### Epidemiologic Characterization of the Outbreak

During January 28–May 5, 2019, a total of 301 meningitis cases were reported from 6 districts in the Est (Diapaga, Gayeri, Pama, and Bogandé) and Sahel (Sebba and Dori) regions through Burkina Faso’s national meningitis CBS, corresponding to a cumulative incidence of 17 cases/100,000 population during this 14-week period (Figure 3). Diapaga experienced the highest disease burden during the outbreak; the cumulative incidence was 29 cases/100,000 population. Cumulative incidences per 100,000 population were 27 cases in Sebba, 15 in Pama, 14 in Gayeri, and 6 each in Dori and Bogandé (Figure 2). We calculated all cumulative incidence rates according to suspected cases. Of the total reported cases, 290 (96%) had specimens collected; among those specimens, 286 (99%) were transported to an NRL and tested by PCR or culture. During the outbreak, the total case-fatality rate reported through CBS in the 6 districts was 5.6%. Of the 301 suspected meningitis cases, the pathogen was confirmed for 137 (46%): 103 (75%) cases were caused by NmC, 16 (12%) by *S. pneumoniae*, 13 (9%) by NmX, 3 (2%) by non–type b *H. influenzae*, and 2 (1%) by *H. influenzae* type b (Tables 1, 2).

**Table 1:** Number of PCR-confirmed bacterial meningitis cases during January 28–May 5, 2019, in study of *Neisseria meningitidis* serogroup C clonal complex 10217 expansion in Burkina Faso*

Bacteria	No. (%) cases
*Neisseria meningitidis*	
Serogroup A	0
Serogroup B	0
Serogroup C	103 (75)
Serogroup W	0
Serogroup X	13 (9)
Serogroup Y	0
*Streptococcus pneumoniae*	16 (12)
*Haemophilus influenzae*	
Type b	2 (1)
Non-b	3 (2)
Total	137

*Cases were from Diapaga, Sebba, Dori, Bogandé, Sebba, and Gayeri districts.

**Table 2:** Number of reported cases of meningitis according to causative pathogen during January 28–May 5, 2019, in study of *Neisseria meningitidis* serogroup C clonal complex 10217 expansion in Burkina Faso*

Characteristics	Suspected/probable cases†	Confirmed cases, n = 137			
		NmC	NmX	*Streptococcus pneumoniae*	*Haemophilus influenzae*
Total no. cases	164	103	13	16	5
Patient sex					
F	92 (56)	45 (44)	3 (23)	5 (31)	3 (60)
M	72 (44)	58 (56)	10 (77)	11 (69)	2 (40)
Districts reporting cases					
Diapaga	87 (53)	54 (52)	6 (46)	2 (13)	2 (40)
Sebba	33 (20)	30 (29)	0	1 (6)	0
Dori	13 (8)	2 (2)	6 (46)	2 (13)	0
Bogandé	14 (9)	4 (4)	0	4 (25)	3 (60)
Pama	10 (6)	5 (5)	0	5 (31)	0
Gayeri	7 (4)	8 (8)	1 (8)	2 (13)	0

*Values are no. (%). A total of 301 cases were reported. NmC, *Neisseria* *meningitidis* serogroup C; NmX, *Neisseria meningitidis* serogroup X. †Includes 3 cases that met the definition of a probable case as previously described (*18*).

**Figure 4:**
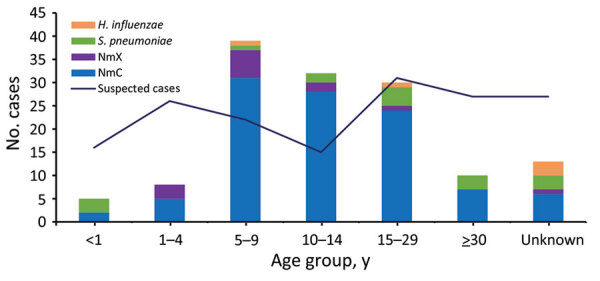
Age distribution of patients with suspected and confirmed meningitis in study of expansion of NmC clonal complex 10217 during meningitis outbreak, Burkina Faso, January 28–May 5, 2019. Data are from districts of Diapaga, Sebba, Dori, Bogandé, Sebba, and Gayeri, Colors indicate the confirmed cause of meningitis; black line indicates number of suspected cases for each age group. The number of suspected cases includes 3 cases that met the definition of a probable case as previously described (*18*). NmC, *Neisseria meningitidis* serogroup C; NmX, *N. meningitidis* serogroup X.

Among the 103 persons with confirmed NmC infection, 83 (86%) persons were 5–29 years of age, representing a narrower age distribution than suspected case-patients, of whom only 50% were in this age range (Figure 4). A preponderance of confirmed NmX was observed among cases reported in Dori; although only 23 (7.6%) suspected cases were reported from Dori, 6 (46%) of the 13 confirmed NmX cases were reported from this district.

### Molecular Typing and Phylogeny

**Figure 5:**
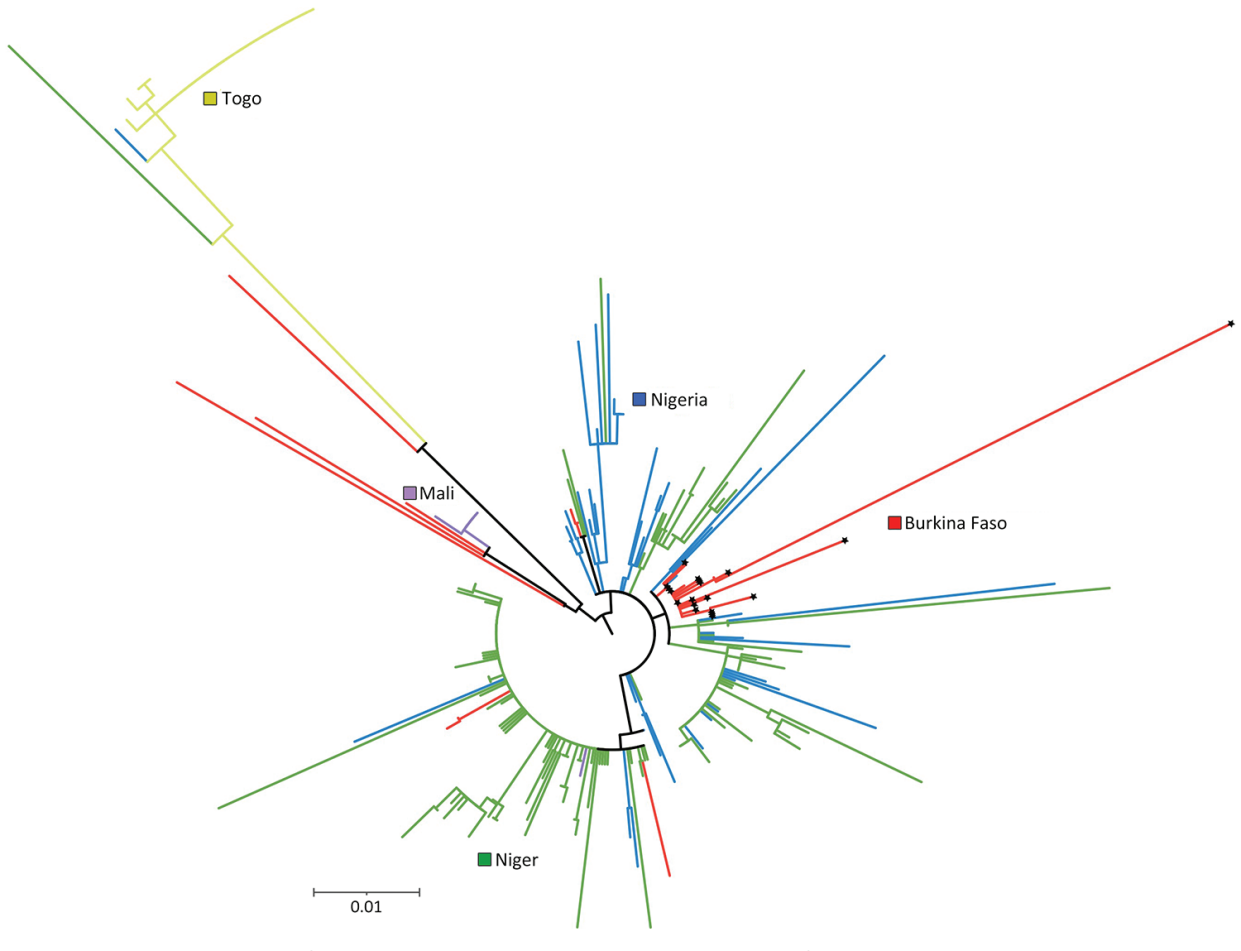
Phylogenetic analysis of *Neisseria meningitidis* clonal complex 10217 isolates from invasive meningitis cases collected during 2012–2019 in Mali, Nigeria, Burkina Faso, Togo, and Niger used in study of expansion of *N*. *meningitidis* serogroup C during meningitis outbreak, Burkina Faso, January 28–May 5, 2019. Colors indicate the major clades/subclades found in each country. Solid black stars on nodes indicate isolates from the Burkina Faso outbreak in 2019. Scale bar indicates nucleotide substitutions per site.

A total of 18 clinical specimens from the outbreak, collected during January 28–May 6 from Diapaga zones 1–3 (9 specimens from zone 1, 6 from zone 2, 3 from zone 3), were whole-genome sequenced at the CDC Bacterial Meningitis Laboratory. The patients from whom the specimens were collected came from 13 different villages. All 18 *N. meningitidis* strains belonged to serogroup C and ST10217, the core ST of CC10217. In addition, all specimens shared the same variant type: PorA type P1.21-15,16; FetA type F1–7; and PorB type 3–463. Phylogenetic analysis (Figure 5) indicated the *N. meningitidis* strain from Burkina Faso shared a common ancestor with ST10217 strains that have been causing disease in Niger and Nigeria since 2013. The strains most closely related to those from Burkina Faso were isolates collected from Niger in 2017.

## Discussion

We report an NmC ST10217 outbreak in Burkina Faso, which occurred during the 2019 epidemic season, that demonstrates the expansion of this epidemic-prone strain into and within Burkina Faso. Despite a history of this strain causing large epidemics, the 2019 outbreak in Burkina Faso remained relatively small, possibly because of the responsive national surveillance system and rapid implementation of reactive vaccination campaigns.

According to the ICG’s performance indicator for timely outbreak response, a country should take <28 days from the time of crossing the meningitis epidemic threshold to implementing a reactive vaccination campaign. Both vaccination campaigns in Diapaga exceeded this indicator; the first vaccination campaign using national vaccine stock in zone 1 was initiated in 6 days, and the second campaign in zones 2, 3, and 4 was initiated 26 days after crossing the epidemic threshold. However, the third vaccination campaign in Sebba and Gayeri was delayed and was implemented 67 days after crossing the epidemic threshold. Key factors affecting vaccination campaign plans in Sebba and Gayeri were geographic barriers and insecurity that made some of the target areas difficult to access. The high proportion of collected specimens, rapid specimen transport to and confirmatory testing at the NRL, and rapid transmission of available surveillance data through STELAB enabled swift outbreak confirmation and coordination of response efforts. Furthermore, the availability of vaccines in the national stockpile likely expedited the particularly rapid response (6 days) for the first vaccination campaign in Diapaga zone 1, highlighting the utility of having decentralized vaccine stockpiles at national and regional levels. This strategy might become more feasible once a sufficient stock of the upcoming NmCV-5 pentavalent conjugate vaccine covering serogroups A, C, Y, W, and X becomes available (*21*). The NmCV-5 vaccine was prequalified in July 2023 and is expected to be available in ICG’s emergency stockpile in early 2024.

Recognizing NmC ST10217’s potential to cause explosive outbreaks and continued expansion, at-risk countries in the region should continue prioritizing, investing, and building a responsive meningitis surveillance and laboratory network to rapidly guide vaccination response in outbreak settings. Smaller focal *N. meningitidis* outbreaks should not be overlooked because a characteristic pattern of meningococcal disease is for a local outbreak to presage a large widespread epidemic (*22*). Before the rollout of MenAfriVac, large epidemic waves caused by NmA were observed over several decades; small, focal outbreaks preceded an explosive epidemic every 5–12 years (*23*,*24*). The major variation in incidence observed with those epidemic waves is believed to be unique to meningococcus (*24*). Thus, despite the smaller magnitude of the 2019 NmC meningitis outbreak compared with those in 2015 and 2017, NmC will likely not disappear from the region without vaccine intervention. NmC continued to be detected in the meningitis belt during 2020–2023 (*25*). Awareness of potential increases in NmC meningitis cases during the next meningitis season will be critical. Although not the dominant pathogens detected, meningitis cases caused by *S. pneumoniae* and *H. influenzae* were also reported during this outbreak. Burkina Faso introduced the *H. influenzae* type b conjugate vaccine in 2006 and the 13-valent pneumococcal conjugate vaccine in 2013. Further analyses of serotype data will help strengthen surveillance and monitoring of invasive disease caused by those bacteria.

The pattern and manifestation of the 2019 meningitis outbreak during the dry season, which is dominated by the Harmattan winds, is consistent with the typical seasonality of meningococcal meningitis outbreaks in the region (*26*–*28*). Similarly, the age distribution of patients with confirmed NmC infections did not differ much from what has been typically observed for meningococcal disease; 86% of patients with confirmed NmC were 5–29 years of age (*11*). However, the difference in age distribution between confirmed NmC and suspected cases during the 2019 outbreak suggests that a substantial percentage of the suspected cases are likely not false negatives but might represent other pathogens or indicate increased care-seeking among certain age groups. The difference is expected because the suspected case definition is designed to have high sensitivity but low specificity, and many other diseases manifest symptoms similar to bacterial meningitis.

Three key limitations affected the analysis of the 2019 NmC infection outbreak. First, the ongoing humanitarian crisis in Burkina Faso caused by clashes between armed extremist groups has resulted in a greater need for resources to implement public health interventions. Security concerns have led to large-scale internal displacement of citizens, as well as health facility closures (*29*). During the 2019 meningitis season, this crisis affected several of the areas that reported NmC cases. The humanitarian crisis might have reduced the likelihood of symptomatic persons effectively seeking healthcare and, thus, reduced the number of reported cases. In addition, incidence estimates relied on population projections from 2010, which were unable to account for major population movements, such as those caused by internal displacement. Second, data discrepancies between STELAB and TLOH were observed. In TLOH, 292 suspected cases were reported during the outbreak (compared with 301 in CBS), and a case-fatality rate of 8.2% was reported (compared with 5.6% in CBS). Data discrepancies and potential underreporting suggest that outbreak characteristics and cases reported in this study are not fully representative of all NmC cases that occurred during the outbreak. Third, administrative vaccine coverage estimates, which were used to approximate reactive vaccination coverage after the outbreak, can be biased because of inaccurate numerators or denominators (*30*). Coverage estimates for all 3 vaccination campaigns conducted during this outbreak are likely overestimates because inflated numerators caused by population movement were heavily affected by the ongoing humanitarian crisis.

In conclusion, meningococcal meningitis remains a serious public health threat within the meningitis belt of Africa. The 2019 NmC outbreak in Burkina Faso shows that a responsive national surveillance system and laboratory network providing timely, spatially explicit case-level data can strengthen outbreak monitoring, response efforts, and tracking of bacteria strains across the region. Detection of NmC ST10217, the strain responsible for previous large-scale epidemics in Niger and Nigeria and the cause of the 2019 Burkina Faso meningitis outbreak, reaffirms the capacity for novel strains to cross geographic boundaries. Preventive mass vaccination campaigns using a long-lasting meningococcal conjugate vaccine have proved effective in dramatically reducing disease, as demonstrated by the national rollout of the MenAfriVac vaccine across the belt. Since the 2019 outbreak in Burkina Faso, 3 consecutive NmC outbreaks have occurred in neighboring Niger during the 2020–2023 epidemic seasons (*25*). The NmC outbreak in Niger during the 2022–23 season also spread to neighboring districts in Nigeria (*31*). Continued NmC outbreaks documented in the belt since the 2019 outbreak and circulation of other *N. meningitidis* non-A serogroups in the region indicate a crucial need for the NmCV-5 vaccine (*21*). In particular, stocking this vaccine at the regional or national level would help ensure that vaccines are immediately ready for use in regional vaccination campaigns when needed. This vaccine strategy could substantially reduce disease caused by non-serogroup A meningococcal pathogens and serves as a key step toward eliminating meningitis outbreaks in the meningitis belt.

Appendix Additional information for expansion of *Neisseria meningitidis* serogroup C clonal complex 10217 during meningitis outbreak, Burkina Faso, 2019.
